# P53-regulated autophagy and its impact on drug resistance and cell fate

**DOI:** 10.20517/cdr.2020.85

**Published:** 2021-03-19

**Authors:** Daeun Shim, Lei Duan, Carl G. Maki

**Affiliations:** Department of Cell and Molecular Medicine, Rush University Medical Center, Chicago, IL 60612, USA.

**Keywords:** Autophagy, histone methylation, metabolism

## Abstract

Wild-type p53 is a stress-responsive transcription factor and a potent tumor suppressor. P53 inhibits the growth of incipient cancer cells by blocking their proliferation or inducing their death through apoptosis. Autophagy is a self-eating process that plays a key role in response to stress. During autophagy, organelles and other intracellular components are degraded in autophagolysosomes and the autophagic breakdown products are recycled into metabolic and energy producing pathways needed for survival. P53 can promote or inhibit autophagy depending on its subcellular localization, mutation status, and the level of stress. Blocking autophagy has been reported in several studies to increase p53-mediated apoptosis, revealing that autophagy can influence cell-fate in response to activated p53 and is a potential target to increase p53-dependent tumor suppression.

## ATGs and mTORC1 control autophagy

Three different forms of autophagy have been described thus far, macroautophagy, microautophagy, and chaperone-mediated autophagy. Macroautophagy involves de novo synthesis of double-membrane vesicles to sequester cellular cargo and transport the cargo to lysosomes. Microautophagy describes a process of lysosomal membrane invagination to directly capture cargo. Chaperone mediated autophagy uses chaperones to identify a certain pentapeptide motif of a cargo and directly translocate it across the lysosomal membrane^[1]^. Despite these differences, the different autophagy mechanisms accomplish the same goal of recycling cellular materials and aiding cellular survival. Macroautopahgy is the best studied due to its link to lung and heart diseases, cancer, diabetes, cystic fibrosis, and other conditions^[2]^. This review will focus on p53 involvement in macroautophagy, which will be referred to as autophagy from this point on. The process of autophagy involves formation and elongation of phagophore membranes, engulfment of cargo (cell proteins and organelles) by phagophore membranes to form autophagosomes, fusion of the autophagosomes with lysosomes to form autophagolysosomes, and degradation of the cargo by lysosomal proteolytic enzymes in a low pH environment^[1,3,4]^. Tightly controlled de novo synthesis of autophagosomes is not entirely understood. However, the products of over 20 autophagy-related genes (ATGs) are involved in this process including ATG1 which is part of the ULK1 complex which initiates autophagy by promoting phagophore membrane formation, ATG5-ATG12-ATG16 and LC3B/ATG8 proteins that promote expansion of phagophore membranes to form autophagosomes, p62/sequestosome and related proteins that promote engulfment of selective cargo, and SNARE proteins that promote fusion of autophagosomes with lysosomes^[5]^.

A key regulator of autophagy is the mammalian target of rapamycin complex 1 (mTORC1).

mTORC1 responds to nutrient and energy levels to regulate cell growth and autophagy. In a nutrient-replete environment, active mTORC1 blocks autophagy by phosphorylating and inhibiting factors required for autophagy initiation like ATG13, ULK1, and focal adhesion kinase interacting protein of 200kDa (FIP200)^[6-8]^. At the same time, mTORC1 promotes protein translation and cell growth by phosphorylating factors such as 4EBP1 and S6K. In contrast, a decrease in energy (ATP) levels causes the intracellular AMP/ATP ratio to increase. This increase activates AMP-activated kinase (AMPK) to phosphorylate and activate TSC2, which then forms a complex with TSC1 to inhibit mTORC1. The inhibition of mTORC1 inhibits cell growth and activates autophagy. Low nutrient levels can also inhibit mTORC1^[9]^. Sancak *et al.*^[10]^ reported a portion of mTORC1 is localized at the lysosome where it can carry out amino acid sensing. Amino acid withdrawal inhibited mTORC1 and displaced it from the lysosome, resulting in a subsequent reduction in cell growth and an increase in autophagy. In sum, mTORC1 regulates cell growth and autophagy appropriate to intracellular energy and nutrient levels^[11]^.

## p53 regulation of autophagy

The effect of p53 on autophagy appears to depend on its subcellular localization, mutation status, and the level of stress. Thus, wild-type p53 induced by therapy agents or in response to stress can promote autophagy, while p53 under physiologic (non-stressed) conditions has been reported to inhibit autophagy. Further, cytoplasmic p53 and cancer-derived p53 mutants that localize predominantly in the cytoplasm also inhibit autophagy. In the following sections we will describe various ways in which wild-type p53 can promote autophagy. We will then summarize the findings that cytoplasmic, mutant, and wild-type p53 under non-stressed conditions can inhibit autophagy.

## Wild-type p53 can promote autophagy through mTORC1

One of the ways in which wild-type p53 can promote autophagy is by activating expression of genes whose protein products directly or indirectly inhibit mTORC1^[12,13]^. mTORC1 is activated downstream of PI3K/AKT in multiple receptor tyrosine kinase (RTK) signaling pathways. AKT activates mTORC1 by phosphorylating and inhibiting TSC2^[14]^. P53 can inhibit AKT activation downstream of RTKs by promoting expression of factors such as PTEN, a lipid phosphatase that counteracts PI3K activity^[15]^. P53 can also inhibit mTORC1 by promoting expression of genes in the AMPK energy sensing pathway. These include the SESN1 and SESN2 genes (whose protein products activate AMPK), the *AMPKb* gene, and the gene encoding TSC2^[16-18]^. Additionally, P53 can inhibit mTORC1 by activating expression of Ddti4/REDD1, a protein that inhibits mTORC1 in a TSC1/TSC2-dependent manner^[19,20]^. In sum, p53 can inhibit mTORC1 and thus induce autophagy by promoting expression of factors that inhibit PI3K/AKT signaling (PTEN) and activate or participate in the AMPK energy sensing pathway (i.e., SESN1/2, AMPKb, TSC2, and Ddti4/REDD1).

## Direct transcriptional activation of autophagy-related genes by nuclear p53

In addition to regulating autophagy through mTORC1, as described above, wild-type p53 can also promote autophagy through direct activation of various ATGs and autophagy-related genes. DRAM1 was one of the first autophagy-related factors found to be transcriptionally activated by p53. DRAM1 was identified by Crighton *et al*.^[21]^ in a screen for genes that are activated by p53. DRAM1 is a lysosomal protein involved in the acidification of lysosomes and activation of lysosomal enzymes. Some studies indicate that DRAM1 is required for p53 to promote autophagy and required for p53-mediated apoptosis. This connection to apoptosis suggests p53-mediated autophagy through DRAM1 may contribute to tumor suppression by p53. Later studies showed p53 regulates mRNA levels for the key autophagy regulator LC3B in chronically starved cells^[22]^. Notably, these studies suggested p53 regulates LC3B mRNA processing at a post-transcriptional level. Still, other studies showed that multiple autophagy-related genes are direct transcriptional targets of p53 in addition to DRAM1. An example is a study by Kenzelmann Broz *et al*.^[23]^ in 2013. In their study, the authors combined p53 ChiP-seq with RNA-seq to identify genes that are directly bound by p53 in response to DNA damage and regulated in a p53-dependent manner. The analysis identified a number of autophagy-related genes that are direct targets of p53 including genes that encode upstream regulators of autophagy (e.g., TSC2), autophagy core machinery (e.g., ULK1, ULK2, ATG2b, 4a, 4c, 7, and 10), and lysosomal proteins [e.g., Vamp4]. Interestingly, they also found that autophagy deficiency increased Ras-induced transformation in MEFs, a process that is normally suppressed by p53. The results supported the idea that p53-mediated autophagy suppresses transformation and thus contributes to p53-mediated tumor suppression. To date, at least 15 ATG and autophagy-related genes have been identified as direct transcriptional targets of p53^[12,23,24]^.

## Involvement of p53 in pro-autophagic histone modification

Methylation of lysine residues on histone H3 represents an active or repressive state of gene transcription depending on the specific lysine that is methylated and the degree of methylation. Thus, H3K4me3 and H3K79me2/me3 methylations are associated with active transcription while H3K9me3, H3K27me3, and H4K20me3 methylations are associated with silenced transcription^[25-29]^. H3K36me3 is typically found in the bodies of actively transcribed genes but is also detected in silenced heterochromatin^[26,30]^. Recent studies indicate that ATG genes and subsequent autophagy are under epigenetic control by histone methylation. G9A, a H3K9 methyltransferase, was shown to directly repress the genes involved in autophagosome formation under normal conditions. Artal-Martinez de Narvajas *et al.*^[31]^ reported that when cells are nutrient-deprived, G9A dissociates from chromatin leading to reduced histone H3K9me2 levels and increased H3K9ac levels. In this relaxed chromatin state, transcription of ATGs such as LC3B, WIPI1, DOR, and p62, are promoted. In another study, it was reported that pharmacological inhibition of G9A by BIX01294 increases LC3B mRNA and protein expression, supporting the idea that LC3B gene expression is regulated by histone methylation status^[32]^. Further demonstrating the role of histone methylation state in autophagy regulation, inhibition of the H3K27 methyltransferase EZH2 (subunit of PRC2 methylation complex) by endogenous miR-92b was reported to promote autophagy when MCF7 and MDA-MB-453 breast cancer cells were subjected to starvation and rapamycin treatment^[33]^.

Recent studies suggest p53 can regulate the expression of histone modifying enzymes, including histone lysine demethylases, as a mechanism to control autophagy and cell survival^[34,35]^. Nutlin-3a (Nutlin) is a small molecule MDM2 antagonist and activator of p53. Cancer cells with MDM2 gene amplification are especially sensitive to Nutlin-induced apoptosis while MDM2 non-amplified cells are resistant to apoptosis but undergo cell cycle arrest. In our lab, we used Nutlin to activate p53 and examined the impact of p53 activation on histone methylation, ATG gene expression, and autophagy. H3K9me3 and H3K36me3 were reduced in MDM2 non-amplified cell treated with Nutlin, and this was coincident with increased expression of various ATG genes (including ULK1 and ATG16L) and increased autophagy flux. H3K9me3 and H3K36me3 are targets for demethylation by Jumonji-domain demethylases, and p53 activates transcription of the Jumonji-domain histone demethylase JMJD2b (also called KDM4b). We therefore asked if JMJD2b was required for the changes in histone methylation and autophagy that we observed in these Nutlin-treated cells. Knockdown or pharmacologic inhibition of JMJD2b prevented the reduction in histone methylation observed in Nutlin-treated cells and blocked the increase in ATG gene expression. Most importantly, knockdown or inhibition of JMJD2b, or treatment with the autophagy inhibitor bafilomycin A, sensitized the MDM2 non-amplified cells to Nutlin-induced apoptosis^[35]^. The results support a model in which p53 induction of JMJD2b leads to a reduction in repressive histone methylations and a subsequent increase in ATG gene expression and pro-survival autophagy.

## p53-mediated metabolic shift regulates autophagy: a role for MDM2

Metabolism and autophagy are tightly linked. Cancer cells often have an altered metabolism that includes an increased dependency on glycolysis and a relative reduction in oxidative phosphorylation compared to normal cells. Activated p53 attempts to restore “normal” metabolism to cancer cells by reducing glycolysis and increasing oxidative phosphorylation. This function of p53 is carried out through multiple mechanisms, including transcriptional regulation by p53 of a large set of its metabolic target genes as well as through non-transcriptional control of mitochondrial functions that promote the activity of the electron transport chain^[36,37]^. MDM2 inhibits p53 but also has p53-independent functions through which it can promote tumorigenesis. One of these functions was highlighted in a recent study that showed MDM2 is recruited to chromatin independent of p53. ChIP-seq analysis identified 159 genes upregulated by MDM2 binding. Further studies showed MDM2 is recruited to target gene promoters by binding the ATF3/4 transcription factor. MDM2 target genes were enriched for those involved in serine, glycine, glutamine, and cysteine metabolism, and serine or glycine deprivation increased MDM2 chromatin binding at target genes to sustain serine/glycine biosynthesis and promote tumor growth^[38]^.

Our studies with Nutlin treatment in MDM2-amplified and non-amplified cancer cells supports the idea that metabolism affects autophagy in p53-activated cells and that MDM2 plays a role in this process. As mentioned earlier, Nutlin treatment blocks autophagy and promotes apoptosis in MDM2-amplified cancer cells but promotes autophagy in MDM2 non-amplified cells that are resistant to apoptosis. In our studies we found glycolysis is also inhibited in MDM2 amplified cells treated with Nutlin but not inhibited in MDM2 non-amplified cells. This p53-dependent reduction in glycolysis (metabolic switch) in MDM2 amplified cells coincided with repression of ATGs ^(3, 5, 7, 10, 12)^, disrupted autophagosome and autolysosome formation, and decreased autophagy flux^[39]^. In MDM2 non-amplified cells, glucose starvation or treatment with a pharmacologic glycolysis inhibitor blocked autophagy and sensitized the cells to apoptosis by Nutlin. These findings suggested one or more metabolites downstream of glycolysis can maintain autophagy and survival in Nutlin-treated cells. Alpha-ketoglutarate (αKG) is a citric acid cycle metabolite that is produced downstream of glycolysis and that is also an activating cofactor for several histone demethylases. We found αKG levels coincide with autophagy and survival in cells where p53 is activated by Nutlin^[40]^. Specifically, αKG levels were decreased in MDM2 amplified cells treated with Nutlin, coincident with decreased autophagy and increased apoptosis. In contrast, αKG levels were either increased or unchanged in MDM2 non-amplified cells that were treated with Nutlin and that resisted apoptosis. Importantly, treatment of MDM2 amplified cells with a cell-permeable αKG analog restored autophagy and rescued cells from Nutlin-induced killing^[40]^. In total, the results suggested that MDM2 amplification status determines whether αKG levels are decreased or increased/maintained in Nutlin-treated cells and this, in turn, determines autophagy and cell survival. While the exact role of αKG in autophagy has not been clarified, studies in C. elegans found that αKG can inhibit mTORC activity and increase survival^[41]^. Also, as mentioned above, αKG is an activating cofactor for Jumonji-domain histone lysine demethylases, including JMJD2b that is transcriptionally activated by p53 and contributes to p53-mediated autophagy^[42]^. Thus, p53 may increase JMJD2b levels as well as activate cofactor αKG to promote or maintain ATG gene expression and ultimately promote autophagy and cell survival. Finally, it is important to note a recent study that reported a link between p53 and αKG levels in a mouse model of pancreatic ductal adenocarcinoma. In that study, restoration of wild-type p53 activity led to an accumulation of αKG, leading to the epigenetic re-activation of cell differentiation genes^[43]^. While the increase in αKG levels upon p53 restoration are consistent with our own findings in MDM2-non amplified cells, the underlying mechanisms involved in αKG accumulation do not seem to operate in MDM2-amplified cells. A possible explanation for this is that high levels of MDM2 can mediate degradation of SP1, a transcription factor that promotes expression of multiple glycolytic pathway genes. We found that SP1 was degraded in MDM2-amplified cells treated with Nutlin in which MDM2 was induced to very high levels^[44]^. The reduction in SP1 coincided with reduced expression of glycolytic pathway genes and reduced αKG levels. Insofar as αKG is produced downstream of glycolysis, we speculate that high levels of MDM2 in MDM2-amplifed cells treated with Nutlin cause degradation of SP1, and this results in repression of glycolytic pathway genes and a corresponding reduction in αKG.

## Cytoplasmic and mutant p53s inhibit autophagy

The first evidence that cytoplasmic p53 can inhibit autophagy came from Tasdemir *et al*.^[45]^ in 2008. In their study knockout of wild-type p53 or inhibition of p53 by the small molecule pifithrin increased autophagy in various cell lines. Re-expression of wild-type p53 reduced autophagy in cells where the endogenous p53 gene had been deleted. These findings suggested that under physiologic, non-stressed conditions p53 normally inhibits autophagy. Gene expression analysis indicated this effect of wild-type p53 likely occurred in a transcription independent way. The authors therefore examined if inhibition of autophagy was a p53 cytoplasmic function. Forms of p53 that localized exclusively in the cytoplasm (e.g., by deletion of the nuclear localization signal) inhibited autophagy whereas p53s that localized exclusively in the nucleus (e.g., by deletion of the nuclear export signal) did not inhibit autophagy. A proposed model is that wild-type p53 is normally expressed at low levels and at least partially cytoplasmic where it inhibits autophagy. In response to stress, p53 accumulates in the nucleus where it can induce autophagy through the various mechanisms mentioned earlier^[45,46]^.

Cancer-associated mutations in p53 occur in the DNA binding domain and inhibit the ability of p53 to bind DNA and activate transcription. Some mutations confer gain-of-function (GOF) properties on mutant p53 that can increase tumorigenesis. Some p53 mutants localize at least partially in the cytoplasm while others localize in the nucleus. Multiple studies have reported that cancer-associated p53 mutants inhibit autophagy^[47-50]^. Studies from the Kroemer lab reported that p53 mutants that localize in the cytoplasm can inhibit autophagy while mutants that localize in the nucleus cannot^[50]^. This suggested cytoplasmic localization is important for mutant p53s to inhibit autophagy. A small portion of wild-type p53 that is induced by stress can localize in the mitochondria and induce apoptosis through interactions with Bcl-2 family members^[51]^. This raised the possibility that cytoplasmic p53 localized in the mitochondria might inhibit autophagy. However, in the Kroemer study cytoplasmic p53 mutants that lacked the ability to localize in the mitochondria and bind Bcl-2 family proteins could still inhibit autophagy, ruling out that the mitochondrial activity of p53 was involved^[50]^. At least two mechanisms have been described for how cytoplasmic and/or mutant p53s inhibit autophagy. First, Zhou *et al*.^[49]^ reported GOF mutant p53s (but not wild-type p53) can bind and inhibit AMPK. This causes an increase in mTORC1 activity and cell growth and a corresponding decrease in autophagy. Second, Cordani *et al*.^[48]^ reported mutant p53s repress expression of several autophagy-related proteins and enzymes including beclin-1, DRAM, ATG12, and SESN1/2. A model was proposed in which a p50 NFkB/mutant p53 complex was recruited to the promoters of these genes to repress their expression (this second model requires that mutant p53 enter the nucleus). There are at least two possible reasons why it might be advantageous for mutant p53s, including GOF mutants with increased oncogenic activity, to inhibit autophagy. One, autophagy is a catabolic process that is counter-productive to cell growth. Inhibiting autophagy while also increasing mTORC1 activity could be a mechanism by which GOF mutant p53s promote cancer cell growth. Two, while autophagy is generally considered a survival mechanism, excess autophagy can also lead to so-called autophagic cell death. Thus, reducing autophagy may prevent autophagic cell death and this may be a mechanism by which GOF mutant p53s increases cancer cell survival.

## p53-mediated autophagy affects cell fate in response to therapeutic agents and stress

p53 mutation or loss has been linked in several studies to reduced tumor therapy responses and worse patient outcome. GOF mutant p53s can promote chemotherapy and radiation resistance through multiple mechanisms including activating expression of certain miRNAs and therapy resistance genes (e.g., MDR1) and acting as a dominant negative inhibitor of wild-type p53 or p73^[52]^. If autophagy promotes survival, then one might expect autophagy inhibition by mutant p53s could enhance therapy sensitivity. However, it is unclear at present how or if autophagy inhibition by mutant p53s impacts therapy responses. In fact, heightened autophagy has been linked with acquired chemotherapy and drug resistance in cancer cells in multiple studies, including in cancer cells expressing either wild-type or mutant p53^[53-57]^. Autophagy inhibitors in many cases can overcome the acquired chemotherapy and drug resistance in these studies. Thus, heightened pro-survival autophagy appears to be a general feature of chemotherapy and drug-resistant cancers regardless of p53 status.

There is abundant crosstalk between autophagy and apoptosis that can influence chemotherapy and drug sensitivity. Thus, activated caspases can promote cleavage of various ATG proteins to inhibit or reduce autophagy, while autophagy has been reported to inhibit apoptosis at least in part by degrading pro-apoptotic factors such as caspase-8^[58,59]^. Damaged proteins and organelles can be a source of stress signals such as reactive oxygen species with the potential to trigger an apoptotic cascade. Thus, a second mechanism by which autophagy could inhibit or reduce apoptosis is by ridding the cell of damaged organelles and proteins.

### Autophagy can protect cancer cells from p53-mediated apoptosis

What is the evidence that autophagy activated by p53 promotes survival, and what are the mechanisms involved? MDM2 antagonists such as Nutlin and its derivatives are being developed as cancer therapeutics. In our studies, we found p53 induced by Nutlin promotes autophagy in cells that are resistant to Nutlin-induced apoptosis (i.e., U2OS and A549) but inhibits autophagy in MDM2-amplified cells that are sensitive to apoptosis by Nutlin (i.e., SJSA1 and MHM). Autophagy inhibitors chloroquine and bafilomycin A1 have sensitized U2OS and A549 cells to Nutlin-induced apoptosis, demonstrating that the autophagy was protective. Nutlin activated caspase-8 in the MDM2-amplified cells that are sensitive to apoptosis but not in the apoptosis resistant cells. However, co-treatment with agents that reduced autophagy sensitized resistant cells to apoptosis, and this was associated with activation of caspase-8^[39]^. The results suggested p53-mediated autophagy in response to Nutlin may protect cells from apoptosis by degrading and inhibiting pro-apoptotic factors like caspase-8. It seems likely this mechanism could also promote survival in response to other agents that induce apoptosis in a manner that involves caspase-8. The findings of Fitzwalter *et al*.^[60]^ are consistent with our results. Specifically, they found the transcription factor FOXO3a promotes expression of the pro-apoptotic BH3-only protein PUMA and is degraded by autophagy. Inhibiting autophagy stabilized FOXO3a which then promoted high expression of PUMA. In their study, blocking autophagy by Bafilomycin A1 treatment sensitized HCT116 colon cancer cells to Nutlin-induced apoptosis. These studies demonstrated that autophagy can promote survival in response to MDM2 antagonists like Nutlin by promoting degradation of FOXO3a and thus preventing PUMA expression. We hypothesize this mechanism could also promote survival in response to other agents that induce apoptosis in a PUMA-dependent manner.

Others have examined the effect of autophagy in response to radiation and chemotherapy and the involvement of p53 in this response. For example, Seiwert *et al*.^[61]^ examined autophagy in response to DNA double strand breaks (DSBs) induced by ionizing radiation or the bacterial cytolethal distending toxin (CDT) in HCT116 colon cancer cells. They found DSBs induced autophagy dependent on ATM kinase and p53. Importantly, they found the autophagy inhibitor chloroquine sensitized cells to killing by CDT, supporting the idea that p53-dependent autophagy protects cells from agents like ionizing radiation and CDT that induce DSBs. Related to this are studies from the Gerwitz group. In their study they examined radiation-induced autophagy in breast, colon, and lung cancer cell lines that vary in p53 status or had p53 deleted by shRNA. They found that radiation could induce autophagy regardless of p53 status. Interestingly, however, autophagy inhibition sensitized p53 wild-type cells to radiation-induced killing but not cells that lacked wild-type p53^[62]^. These findings raised the possibility that the cytoprotective (survival) effect of autophagy in irradiated cells is dependent on wild-type p53. Alternatively, the results could mean autophagy inhibition sensitizes cells to radiation in a p53-dependent manner. Studies by Zeng *et al*.^[63]^ examined the relationship between autophagy and apoptosis in mismatch repair (MMR) proficient and deficient colon cancer cells treated with the chemotherapy agent 6-thioguanine (6-TG). The authors found that 6-TG treatment induced autophagy dependent on MMR activity and dependent on p53. Knockdown of the critical autophagy regulator ATG5 or pharmacologic inhibition of autophagy sensitized 6-TG treated cells to apoptosis. While the mechanism of how autophagy protects cells from 6-TG was not determined, the results nonetheless indicated p53-mediated autophagy can protect cancer cells from killing by the therapy agent 6-TG^[63]^.

Finally, another possible mechanism by which autophagy could protect cells from p53-induced apoptosis comes from studies of p53 in replication stress. Wild-type p53 is activated in response to replication stress, and recent studies have shown that p53 promotes replication fork processivity that may contribute to its tumor suppressor function^[64]^. In unpublished studies, we have gained evidence that p53 induced by the replication stressor hydroxyurea (HU) promotes autophagy, and that bafilomycin A1 co-treatment sensitizes HU-treated cells to apoptosis. Vanzo *et al*.^[65]^ recently reported that autophagy can help maintain replication forks in response to replication stressors by maintaining nucleotide levels. Based on this, we speculate autophagy may also protect cells from p53-induced killing in response to replication stresses by maintaining nucleotide levels.

### Autophagy can contribute to p53-mediated apoptosis

While the studies described above indicate autophagy can protect cells from p53-mediated death/apoptosis in response to radiation and certain therapy agents, other studies suggest the opposite. One example is the study by Borthakur *et al*.^[66]^ in which they examined autophagy and apoptosis in Nutlin-treated acute myelocytic leukemia (AML) cells. They found Nutlin induces autophagy in AML cells in a manner that appears to involve p53 activation of AMPK and subsequent inhibition of mTORC1. Notably, in their study, autophagy inhibition by Bafilomycin A1 reduced apoptosis in Nutlin-treated AML cells, supporting the idea that autophagy induction contributed to apoptosis^[66]^. Another example is the study by Kenzelmann Broz *et al*.^[23]^, described above, in which ChIP-seq and RNAseq were used to identify autophagy genes regulated by p53 in MEFs treated with the DNA damaging agent doxorubicin. In that study, it was found that p53 bound and activated expression of multiple ATG genes and promoted autophagy in response to doxorubicin treatment. Inhibition of autophagy by ATG5 knockout reduced p53-dependent apoptosis in response to doxorubicin, supporting the idea that p53-mediated autophagy contributes to doxorubicin induced killing^[23]^. Yet another example is the study by Gao *et al*.^[67]^ In their study, U2OS osteosarcoma cells were treated with camptothecin or etoposide. The authors found that p53 induced autophagy in response to both treatments, and that inhibiting autophagy rescued the cells from camptothecin-induced killing.

## Conclusion

There are several reports that demonstrate autophagy can protect cells from p53-mediated apoptosis and cancer cell killing in response to radiation, chemotherapy, and small molecule MDM2 antagonists. These findings would support the potential for combining autophagy inhibitors with therapy agents that stabilize and/or activate p53 to improve cancer cell responses. However, there is also evidence that autophagy can contribute to p53-mediated killing in cells exposed to MDM2 antagonists and certain therapeutic drugs. Thus, the impact of autophagy on p53-mediatd apoptosis and cancer cell killing in response to radiation and therapeutic drug treatment is likely cell-type and context dependent. A better understanding of how autophagy regulates cell fate in response to activated p53 will be required for future consideration of autophagy inhibitor usage in cancer patients.

**Figure 1:**
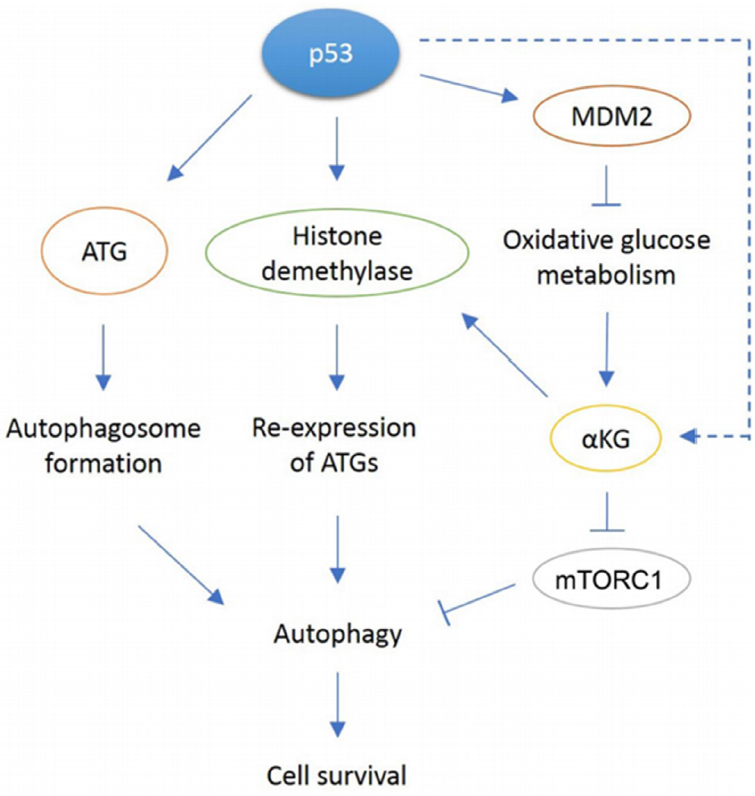
In response to cellular stress, p53 can promote autophagy through various mechanisms. p53 can directly bind the conserved binding site in ATG gene promoters and transcribe proteins required for autophagosome formation. p53 can also induce transcription of JMJD2B demethylase that removes methylation on histone H3, allowing re-expression of previously repressed ATGs. Another proposed mechanism is through p53-mediated oxidative metabolism. Through activation of multiple target genes, p53 can shift metabolism away from glycolysis to favor oxidative metabolism instead. The reduction in glycolysis has been observed only in MDM2 amplified tumor cells. A resulting metabolite, αKG is a cofactor for JMJD2B, so it may be possible to play a role in histone modification that leads to re-expression of ATGs. Our paper showed αKG levels decreased in MDM2-amplified cells treated with Nutlin but increased in response to Nutlin in MDM2 non-amplified cells through an unknown mechanim (dotted arrow). Also, αKG may be involved in mTORC inhibition as observed in C elegans

P53 can promote autophagy through multiple mechanisms. These mechanisms include direct transcriptional activation of ATG genes by p53, and indirect regulation of these genes by p53 through alterations in glycolysis, histone methylation, and α-KG levels [Figure 1]. While Figure 1 depicts autophagy as a general survival mechanism, it is important to note that autophagy can also have a tumor suppressive role that appears dependent, at least in part, on cancer stage^[68]^. In response to cancer therapy agents, tumor cells can manipulate autophagy to promote tumor survival^[69]^. While more work is needed, inhibition of autophagy may be considered as a potential treatment adjuvant in patients who display chemo and/or therapy resistance.

## Declarations

### Copyright

© The Author(s) 2021.
